# Comparing the Safety and Effectiveness of PCL and PLLA Injections for Nasolabial Fold Correction

**DOI:** 10.1111/jocd.70414

**Published:** 2025-08-28

**Authors:** Kaixuan Hu, Shanqing Wang

**Affiliations:** ^1^ Plastic Surgery Department Quzhou Kecheng Dainiel Plastic Surgery Outpatient Quzhou City China; ^2^ Department of Dermatology, School of Medicine Rui Jin Hospital, Shanghai Jiao Tong University Shanghai City China

**Keywords:** aesthetic improvement, nasolabial folds, patient satisfaction, polycaprolactone, poly‐l‐lactic acid, wrinkle severity

## Abstract

**Background:**

Nasolabial folds (NLF) deepen with age, necessitating aesthetic treatments. Polycaprolactone (PCL) and poly‐l‐lactic acid (PLLA) are commonly used fillers. This study compares their safety and efficacy for NLF correction.

**Methods:**

Participants aged 30–60 years were retrospectively reviewed after undergoing NLF correction with PCL (*n* = 65) or PLLA (*n* = 71) from January 2023 to December 2023. Primary outcomes included changes in the Nasolabial Fold Wrinkle Severity Rating Scale (WSRS) at baseline and 3, 6, and 12 months post‐treatment. Secondary outcomes assessed patient satisfaction via the FACE‐Q scale, Global Aesthetic Improvement Scale (GAIS), and safety profiles including side effects and adverse events.

**Results:**

At 3 months, PCL exhibited significantly greater wrinkle reduction (WSRS score: 3.08 ± 0.37) compared to PLLA (3.27 ± 0.41; *p* = 0.007). This trend persisted at 6 months (PCL: 2.23 ± 0.61 vs. PLLA: 2.49 ± 0.75; *p* = 0.03) and 12 months (PCL: 1.92 ± 0.55 vs. PLLA: 2.17 ± 0.61; *p* = 0.016). PCL also showed higher significant improvement rates on GAIS at 3 and 6 months. Patient satisfaction (FACE‐Q) was higher in the PCL group at both 6 and 12 months (*p* < 0.05). Safety profiles were comparable, with no significant differences in injection site reactions or adverse events.

**Conclusion:**

PCL injections demonstrated superior effectiveness in reducing NLF wrinkle severity and achieving higher patient satisfaction compared to PLLA injections over a 12‐month period. Both materials exhibited comparable safety profiles, suggesting the suitability of PCL as a preferable option for NLF correction in cosmetic dermatology.

## 1 Introduction

Nasolabial folds (NLF) are a prominent and common facial feature that deepens with age due to the loss of skin elasticity, subcutaneous fat, and collagen [1]. These creases, extending from the sides of the nose to the corners of the mouth, can impart an aged or tired appearance, prompting many individuals to seek corrective treatments [2]. The aesthetic correction of these folds has thus become a focal point of non‐surgical facial rejuvenation [3]. Injectable fillers have emerged as a popular option for patients desiring less invasive alternatives to traditional surgical procedures [4].

Globally, the demand for dermal fillers has seen a substantial rise, driven by the growing emphasis on aesthetic enhancement and the preference for minimally invasive cosmetic procedures [5]. According to the American Society of Plastic Surgeons (ASPS), soft tissue filler procedures increased by 2% from 2019 to 2020, with over 3.4 million procedures performed in 2020 alone [6]. This upward trend is mirrored globally, reflecting the widespread appeal of these treatments [7]. Among these, the correction of NLF remains one of the most common indications for dermal filler use [8].

Polycaprolactone (PCL) and poly‐l‐lactic acid (PLLA) are two of the most commonly used biodegradable materials in dermal fillers, each possessing unique properties that influence their clinical effects [8, 9]. PCL is a long‐lasting synthetic polymer that provides structural support and induces collagenesis [10]. Its degradation process takes place over a more extended period, often up to 2–3 years, which can result in sustained volumization and wrinkle correction [11]. On the other hand, PLLA is a biocompatible, biodegradable polymer that primarily acts as a collagen stimulator. PLLA particles initiate a robust inflammatory response that promotes fibroblast activation and collagen production, leading to gradual and less immediate volume restoration compared to more cohesive fillers [12].

The clinical efficacy and safety profiles of PCL and PLLA have been the subject of numerous studies [13]. However, comparative data specifically focusing on their use in NLF correction remain limited [13]. Considerable variations exist in their mechanisms of action, duration of effects, and potential side effects, making it essential to rigorously evaluate their relative performance in a controlled setting [14].

This study aims to address this gap by retrospectively comparing the safety and effectiveness of PCL and PLLA injections for NLF correction. We hypothesize that the distinct physical and biological characteristics of these materials will result in different clinical outcomes and patient satisfaction levels, thereby informing best practices for their application in aesthetic dermatology.

## 2 Materials and Methods

### 2.1 Study Design

This retrospective cohort study enrolled patients who underwent nasal labial fold correction at our institution between January 2023 and December 2023. The patients were divided into two groups based on the corrective materials used: PLLA (*n* = 65) and PCL (*n* = 71). Physicians followed individual patient needs in material selection (such as the desired duration of effect and economic factors), but the procedural techniques (such as injection angles and layers) strictly adhered to the product instructions and international consensus guidelines. The consistency of the physicians' experience and techniques in both groups has been verified through preoperative training records.

This study was approved by the Ethics Committee of Quzhou Kecheng Dainier Plastic Surgery Outpatient in accordance with regulatory and ethical guidelines pertaining to retrospective research studies and conducted in accordance with the Declaration of Helsinki. Informed consent was waived for this retrospective study due to the exclusive use of de‐identified patient data, which posed no potential harm or impact on patient care.

### 2.2 Eligibility Criteria

Inclusion criteria encompassed subjects aged 30–60 years of any gender with identical bilateral NLF WSRS grades of 3 to 4 and a willingness to undergo corrective treatment for NLF, who demonstrate good compliance and have reasonable expectations of cosmetic surgical outcomes, adhering to study procedures until clinical trial completion. Exclusion criteria included individuals with active facial infections or inflammation, severe organic or mental disorders, malignant skin tumors, participation in other clinical trials or phototherapy, and injectable treatments in the targeted area within the past 6 months, a history of facial surgery, permanent filler injections in the NLF, or fat grafting, as well as pregnant or breastfeeding women, those planning pregnancy, and individuals with a propensity for scarring.

### 2.3 Surgical Methods

Before starting treatment, all patients underwent a comprehensive examination, encompassing their medical history and current medications. Pretreatment photographs were taken to establish their WSRS (Wrinkle Severity Rating Scale) ratings.

For the PLLA Group, injectable PLLA (Löviselle, Changchun Synthe Biological Materials Co. Ltd., Registration No. 20213130276) was prepared by mixing 5 mL of sterile water with the powder and allowing it to hydrate for at least 2 h before injection. The injections were administered using a 27G blunt needle into the shallow and intermediate subcutaneous layers. Prior to the injection, a topical cream, Compound Lidocaine Cream (Beijing Ziguang Pharmaceutical Industry Co. Ltd., Beijing, China) was applied to the NLF, and the reconstituted product did not contain any anesthetics. Following the initial injection session, follow‐up evaluations were conducted every 4–6 weeks to determine if further correction was necessary.

For the PCL injections (Ellansé, Sinclair, a subsidiary of Huadong Medicine, Registration No. 20213130100), a 25‐G blunt needle was used to deliver the substance into the mid‐to‐deep dermis at an angle of approximately 30°, parallel to the length of the fold. The aim of the initial injections was to achieve suboptimal augmentation of the folds. The treated areas were gently massaged to conform to the surrounding tissue if needed. If swelling occurred immediately after the injection, an ice pack was applied temporarily to the affected area. Patients were advised to avoid excessive sun exposure, UV lamps, and extreme cold weather until the initial redness and swelling had subsided.

### 2.4 Data Collection

Data collection encompassed various aspects of patient information and outcomes: general patient demographics were recorded, including age, gender, wrinkle duration, past cosmetic procedures, Fitzpatrick skin type, previous filler use, residence, and employment status. Surgical and recovery details, such as surgery duration, time to return to work, swelling time, post‐injection discomfort, and pain medication requirements, were documented. NLF wrinkle grades at baseline and at 3, 6, and 12 months post‐treatment were evaluated, along with overall facial aesthetic improvements at 3 and 6 months post‐procedure. Additionally, FACE‐Q aesthetic scale scores were noted at baseline and at 6 and 12 months post‐treatment. Safety outcomes were monitored by recording injection site reactions, nodule formation, discoloration, and infections, while adverse events such as granuloma formation, vascular occlusion, hypersensitivity reactions, and bruising were also meticulously tracked.

### 2.5 Nasolabial Fold WSRS

The Nasolabial Fold Wrinkle Severity Rating Scale (WSRS) categorizes wrinkles into five grades: Grade 1 indicates no visible wrinkles, only continuous skin folds; Grade 2 represents mild wrinkles, more noticeable when smiling, with slight indentations; Grade 3 signifies moderate wrinkles that are visible at rest but disappear when the skin is stretched; Grade 4 describes long and deep wrinkles, prominent even at rest, with < 2 mm visible when stretched; and Grade 5 denotes extremely deep and long wrinkles that severely affect facial appearance, with 2–4 mm visible upon stretching.

### 2.6 Global Aesthetic Improvement Scale (GAIS) Descriptions

The GAIS describes the improvement in overall facial aesthetics as follows: “Very Significant Improvement” indicates achieving the optimal aesthetic outcome post‐implant; “Significant Improvement” denotes a substantial enhancement in appearance compared to the original state, though not optimal; “Moderate Improvement” refers to noticeable appearance enhancement; “No Improvement” means little to no difference in appearance compared to the initial state; and “Worsening” signifies a deterioration in appearance from the original condition.

### 2.7 Patient Satisfaction Assessment Using FACE‐Q

Patient satisfaction was measured using two subscales from the FACE‐Q questionnaire: Satisfaction with Facial Skin and Satisfaction with Nose. Each subscale consists of 12 questions, rated on a 4‐point Likert scale ranging from Very Dissatisfied (1) to Very Satisfied (4). The raw scores (12–48) were converted into a scale ranging from 0 (worst) to 100 (best), with higher scores indicating greater satisfaction. The reliability of each FACE‐Q subscale is robust, with Cronbach's alpha values ranging from 0.88 to 0.951 [15].

### 2.8 Statistical Methods

Data were analyzed using SPSS version 29.0 (SPSS Inc., Chicago, IL, USA). Categorical variables were presented as [*n*(%)]. Categorical variables were analyzed using chi‐square tests. Continuous variables were assessed for normality using the Shapiro–Wilk test. The continuous variable of normal distribution is represented as mean ± standard deviation (mean ± SD). Normally distributed continuous variables were analyzed using *t*‐tests. A two‐sided *p* value of < 0.05 was considered statistically significant.

## 3 Results

### 3.1 Baseline Characteristics of Study Participants

The baseline characteristics of the participants in the PCL group (*n* = 65) and PLLA group (*n* = 71) were comparable (Table 1). The mean age was 45.21 ± 3.57 years in the PCL group and 44.78 ± 3.25 years in the PLLA group (*t =* 0.730; *p =* 0.467). Gender distribution was similar between the groups, with 24 males (36.92%) and 41 females (63.08%) in the PCL group, compared to 28 males (39.44%) and 43 females (60.56%) in the PLLA group (*χ* ^2^ = 0.016; *p =* 0.901). Participants reported a mean duration of wrinkles of 18.56 ± 3.18 months in the PCL group and 19.36 ± 2.57 months in the PLLA group (*t =* 1.611; *p =* 0.11). Prior cosmetic procedures were reported by 20 participants (30.77%) in the PCL group and 20 participants (28.17%) in the PLLA group (*χ* ^2^ = 0.021; *p =* 0.885). Fitzpatrick skin types I–VI were similarly distributed, with no significant variation between groups (*p =* 0.91). Previous filler use was comparable, with 15 participants (23.08%) in the PCL group and 18 participants (25.35%) in the PLLA group (*χ* ^2^ = 0.012; *p =* 0.913). Urban versus rural residence and employment status distributions were analogous across both groups (*p =* 0.648 and *p =* 0.862, respectively).

**TABLE 1:** Baseline characteristics of study participants.

Parameter	PCL group (*n* = 65)	PLLA group (*n* = 71)	*t*/*χ* ^2^	*p*
Age (years)	45.21 ± 3.57	44.78 ± 3.25	0.730	0.467
Gender (male/female)	24 (36.92%)/41 (63.08%)	28 (39.44%)/43 (60.56%)	0.016	0.901
Duration of wrinkles (months)	18.56 ± 3.18	19.36 ± 2.57	1.611	0.11
Prior cosmetic procedures	20 (30.77%)	20 (28.17%)	0.021	0.885
Fitzpatrick skin type (I–VI)	21 (32.31%)/22 (33.85%)/10 (15.38%)/8 (12.31%)/4 (6.15%)	23 (32.39%)/21 (29.58%)/15 (21.13%)/9 (12.68%)/3 (4.23%)	None	0.91
Previous filler use (Y/N)	15 (23.08%)/50 (76.92%)	18 (25.35%)/53 (74.65%)	0.012	0.913
Urban/rural residence (%)			0.209	0.648
Urban	49 (75.38%)	50 (70.42%)		
Rural	16 (24.62%)	21 (29.58%)		
Employment status			0.297	0.862
Employed (%)	50 (76.92%)	53 (74.65%)		
Unemployed (%)	8 (12.31%)	11 (15.49%)		
Retired (%)	7 (10.77%)	7 (9.86%)		

### 3.2 Duration of the Procedure and Recovery Time

The duration of the procedure, recovery time, and post‐injection experiences were similar between the PCL and PLLA groups (Table 2). The mean procedure duration was 32.54 ± 5.69 min for the PCL group and 32.89 ± 5.75 min for the PLLA group (*t =* 0.349; *p =* 0.728). The average time to return to work was slightly shorter in the PCL group (0.98 ± 0.25 days) compared to the PLLA group (1.05 ± 0.28 days), but the difference was not statistically significant (*t =* 1.635; *p =* 0.104). Both groups reported similar post‐injection discomfort scores, with mean values of 3.45 ± 1.08 for the PCL group and 3.52 ± 1.15 for the PLLA group (*t =* 0.354; *p =* 0.724). The need for pain medication was reported by eight participants (12.31%) in the PCL group and 11 participants (15.49%) in the PLLA group, with no significant difference between the groups (*χ* ^2^ = 0.083; *p =* 0.774). Swelling time was significantly longer in the PCL group (3.03 ± 0.78 days) compared to the PLLA group (3.34 ± 0.82 days) (*t* = 2.249, *p* = 0.026). These results indicate that while most measured parameters did not show significant differences between the two groups, the swelling time was notably longer in the PLLA group, suggesting potential clinical implications for post‐procedure recovery.

**TABLE 2:** Duration of the procedure and recovery time.

Parameter	PCL group (*n* = 65)	PLLA group (*n* = 71)	*t*/*χ* ^2^	*p*
Procedure duration (min)	32.54 ± 5.69	32.89 ± 5.75	0.349	0.728
Time to return to work (days)	0.98 ± 0.25	1.05 ± 0.28	1.635	0.104
Swelling time (days)	3.03 ± 0.78	3.34 ± 0.82	2.249	0.026
Post‐injection discomfort (0–10 scale)	3.45 ± 1.08	3.52 ± 1.15	0.354	0.724
Need for pain medication (%)	8 (12.31%)/57 (87.69%)	11 (15.49%)/60 (84.51%)	0.083	0.774

### 3.3 Nasolabial Fold Wrinkle Severity Rating Scale

**FIGURE 1:**
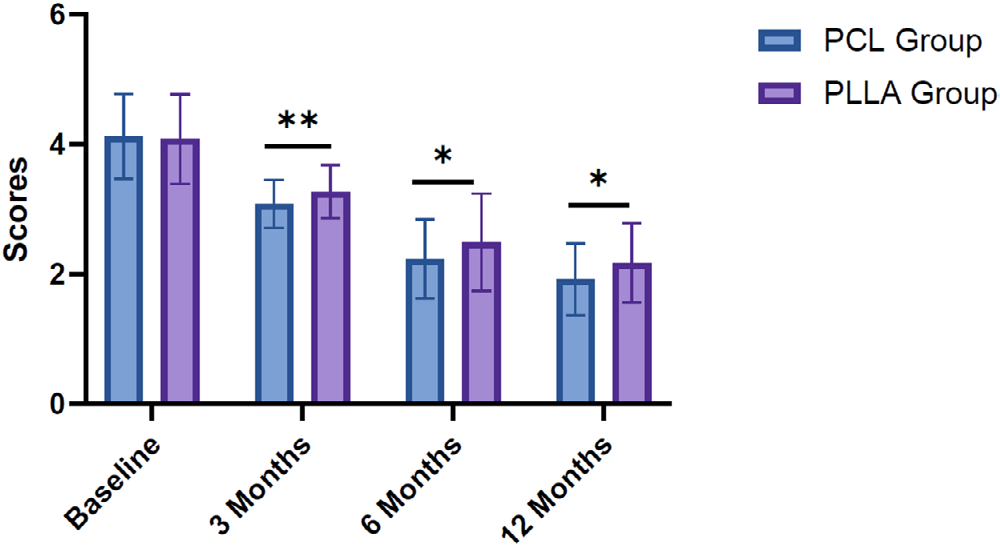
Changes in the severity score of nasolabial folds (WSRS) over time. *p < 0.05, **p < 0.01. Wrinkle Severity Rating Scale (WSRS) was detected at baseline and 3, 6, and 12 months post‐treatment. Polycaprolactone (PCL) group versus poly‐l‐lactic acid (PLLA) group was analyzed by independent sample *t*‐test.

The effectiveness of PCL and PLLA injections for NLF correction was assessed using the Nasolabial Fold Wrinkle Severity Rating Scale at multiple time points (Table 3). At baseline, the mean wrinkle severity scores were comparable between the PCL group (4.12 ± 0.65) and the PLLA group (4.08 ± 0.69) (*t =* 0.372; *p =* 0.711). At 3 months, the PCL group exhibited a statistically significant greater reduction in wrinkle severity (mean score of 3.08 ± 0.37) compared to the PLLA group (mean score of 3.27 ± 0.41) (*t =* 2.745; *p =* 0.007). This trend continued at 6 months, with the PCL group showing a mean score of 2.23 ± 0.61 versus 2.49 ± 0.75 in the PLLA group (*t =* 2.195; *p =* 0.03). At 12 months, the PCL group maintained lower wrinkle severity scores (mean score of 1.92 ± 0.55) compared to the PLLA group (mean score of 2.17 ± 0.61), with the difference remaining statistically significant (*t =* 2.443; *p =* 0.016) (Figure 1).

**TABLE 3:** Global Aesthetic Improvement Scale.

Time point	PCL group (*n* = 65)	PLLA group (*n* = 71)	*χ* ^2^	*p*
3 months				
Very significant improvement	10 (15.38%)	2 (2.82%)	5.192	0.023
Significant improvement	11 (16.92%)	3 (4.23%)	4.63	0.031
Partial improvement	42 (64.62%)	57 (80.28%)	3.452	0.063
No improvement	2 (3.08%)	8 (11.27%)	2.248	0.134
Worsening	0 (0%)/65 (100%)	3 (4.23%)	1.191	0.275
6 months				
Very significant improvement	31 (47.69%)	20 (28.17%)	4.717	0.030
Significant improvement	17 (26.15%)	8 (11.27%)	4.069	0.044
Partial improvement	10 (15.38%)	27 (38.03%)	7.679	0.006
No improvement	6 (9.23%)	11 (15.49%)	0.711	0.399
Worsening	1 (1.54%)	5 (7.04%)	1.307	0.253

### 3.4 Global Aesthetic Improvement Scale

The GAIS scores demonstrated differing degrees of aesthetic enhancement between the PCL and PLLA groups (Table 3). At 3 months, a higher proportion of participants in the PCL group experienced very significant improvement (15.38% vs. 2.82%; *χ* ^2^ = 5.192; *p =* 0.023). Additionally, significant improvement was observed in 16.92% of the PCL group compared to 4.23% of the PLLA group (*χ* ^2^ = 4.63; *p =* 0.031). Partial improvement was noted in 64.62% of the PCL group and 80.28% of the PLLA group (*χ* ^2^ = 3.452; *p =* 0.063), while no improvement and worsening were not statistically significant between both groups (*χ* ^2^ = 2.248; *p =* 0.134 and *χ* ^2^ = 1.191; *p =* 0.275, respectively). At 6 months, 47.69% of the PCL group reported very significant improvement versus 28.17% in the PLLA group (*χ* ^2^ = 4.717; *p =* 0.030), and significant improvement was reported by 26.15% of the PCL group compared to 11.27% of the PLLA group (*χ* ^2^ = 4.069; *p =* 0.044). Conversely, partial improvement was more common in the PLLA group (38.03% vs. 15.38%; *χ* ^2^ = 7.679; *p =* 0.006). The differences in no improvement and worsening at this time point were not statistically significant (*χ* ^2^ = 0.711; *p =* 0.399 and *χ* ^2^ = 1.307; *p =* 0.253, respectively).

### 3.5 FACE‐Q Scale

Patient satisfaction, as measured by the FACE‐Q scale, showed statistically significant differences between the PCL and PLLA groups at 6 and 12 months (Table 4). At baseline, satisfaction with skin (PCL: 42.59 ± 3.59, PLLA: 43.17 ± 3.69; *t =* 0.932; *p =* 0.353) and nose (PCL: 33.81 ± 4.93, PLLA: 32.93 ± 5.28; *t =* 1.010; *p =* 0.314) was not significantly different between the groups. However, at 6 months, satisfaction with skin was significantly higher in the PCL group (66.38 ± 5.83) compared to the PLLA group (63.88 ± 6.74; *t =* 2.322; *p =* 0.022), as was satisfaction with nose (PCL: 75.84 ± 6.09, PLLA: 73.21 ± 5.97; *t =* 2.544; *p =* 0.012). These differences were sustained at 12 months, with the PCL group reporting higher satisfaction with skin (78.91 ± 4.18) compared to the PLLA group (76.49 ± 4.59; *t =* 3.219; *p =* 0.002), and higher satisfaction with nose (PCL: 84.56 ± 6.52, PLLA: 81.27 ± 6.17; *t =* 3.020; *p =* 0.003).

**TABLE 4:** FACE‐Q scale.

Time point	PCL group (*n* = 65)	PLLA group (*n* = 71)	*t*	*p*
Baseline satisfaction with skin	42.59 ± 3.59	43.17 ± 3.69	0.932	0.353
Baseline satisfaction with nose	33.81 ± 4.93	32.93 ± 5.28	1.010	0.314
6 months satisfaction with skin	66.38 ± 5.83	63.88 ± 6.74	2.322	0.022
6 months satisfaction with nose	75.84 ± 6.09	73.21 ± 5.97	2.544	0.012
12 months satisfaction with skin	78.91 ± 4.18	76.49 ± 4.59	3.219	0.002
12 months satisfaction with nose	84.56 ± 6.52	81.27 ± 6.17	3.020	0.003

### 3.6 Safety Outcomes

The safety outcomes for PCL and PLLA injections for NLF correction were similar across both groups, with no statistically significant differences observed (Table 5). Injection site reactions occurred in 15.38% of participants in the PCL group and 16.9% in the PLLA group (*χ* ^2^ = 0; *p =* 0.995). Nodule formation was reported in 4.62% of the PCL group and 0.00% of the PLLA group (*χ* ^2^ = 1.553; *p =* 0.213). Discoloration was noted in 10.77% of participants in the PCL group and 12.68% in the PLLA group (*χ* ^2^ = 0.006; *p =* 0.938). Infection occurred in 1.54% of the PCL group compared to 4.23% in the PLLA group (*χ* ^2^ = 0.175; *p =* 0.676).

**TABLE 5:** Safety outcomes.

Parameter	PCL group (*n* = 65)	PLLA group (*n* = 71)	*χ* ^2^	*p*
Injection site reactions (%)	10 (15.38%)	12 (16.9%)	0	0.995
Nodule formation (%)	3 (4.62%)	0 (0.00%)	1.553	0.213
Discoloration (%)	7 (10.77%)	9 (12.68%)	0.006	0.938
Infection (%)	1 (1.54%)	3 (4.23%)	0.175	0.676

### 3.7 Adverse Events

The occurrence of adverse events following PCL and PLLA injections for NLF correction was low and showed no statistically significant differences between the two groups (Table 6). Granulomas were reported in 3.08% of the PCL group and 1.41% of the PLLA group (*χ* ^2^ = 0.006; *p =* 0.938). Hypersensitivity reactions were observed in 3.08% of the PCL group and 5.63% of the PLLA group (*χ* ^2^ = 0.094; *p =* 0.759). Bruising was reported by 7.69% of participants in the PCL group and 9.86% in the PLLA group (*χ* ^2^ = 0.020; *p =* 0.887).

**TABLE 6:** Adverse events.

	PCL group (*n* = 65)	PLLA group (*n* = 71)	*χ* ^2^	*p*
Granulomas (%)	2 (3.08%)	1 (1.41%)	0.006	0.938
Hypersensitivity (%)	2 (3.08%)	4 (5.63%)	0.094	0.759
Bruising (%)	5 (7.69%)	7 (9.86%)	0.020	0.887

## 4 Discussion

Anatomical aging, characterized by a decrease in subcutaneous fat and collagen, significantly leads to the deepening of the NLF [16]. subcutaneous injection of HA has rapidly developed in the medical beauty market [17]. The biocompatibility, biodegradability, and high water absorption of HA make it the main material for dermal fillers [18]. However, uncross‐linked HA is easily decomposed by enzymes in the physiological environment and subsequently metabolized by the human body, which reduces its tolerance in the skin tissue, thereby limiting the application of HA as a dermal filler [19]. In contrast, stimulating fillers such as polycaprolactone (PCL) and poly‐l‐lactic acid (PLLA) have several advantages over HA fillers. These fillers can induce the production of collagen, providing longer lasting effects and better structural support. PCL and PLLA have been shown to produce sustained volumization and wrinkle correction over a long period of time, thereby reducing the frequency of re treatment [20]. In addition, the collagen stimulating properties of these fillers can bring about a natural appearance with fewer side effects [21]. The results of this retrospective cohort study provide valuable insights into the safety and effectiveness of PCL and PLLA injections used for NLF correction. Our findings contribute to a nuanced understanding of the comparative performance of these two widely used materials in cosmetic procedures. One of the key findings from this study is that PCL injections demonstrated a statistically significant greater reduction in wrinkle severity compared to PLLA injections over the 12‐month follow‐up period. The mechanisms underlying this difference likely pertain to the distinct physicochemical properties of the materials. PCL is a bioresorbable polyester with a longer degradation time, providing a more prolonged tissue stimulation and volumization effect [22, 23]. This sustained stimulation of collagen production could account for the more significant and enduring improvement in wrinkle severity observed in the PCL group [24]. In contrast, PLLA acts primarily as a biostimulatory agent that induces collagen synthesis over a shorter period, potentially explaining the less substantial wrinkle reduction over time [25]. PCL (polycaprolactone) typically degrades over a period of up to 2 years, while PLLA (poly‐l‐lactic acid) degrades within a range of several months to two years [26].

The properties of PCL contributing to its extended persistence in tissue could be driving the more pronounced aesthetic improvements as captured by the GAIS [27, 28]. At both 3 and 6 months post‐treatment, a higher proportion of patients in the PCL group reported very significant improvements compared to the PLLA group. These outcomes suggest that PCL's longer‐lasting structural support within the dermal layers may better sustain facial volume and contour enhancements over time. Furthermore, PCL's ability to withstand enzymatic degradation for up to 2–3 years allows for a more gradual and sustained collagen remodeling process, translating to prolonged aesthetic benefits [29].

Patient satisfaction, measured using the FACE‐Q scale, indicated higher satisfaction levels in the PCL group at both 6 and 12 months post‐treatment. This corroborates with the clinical improvements in wrinkle severity and aesthetic appearance noted with PCL injections. The higher patient satisfaction likely stems from both the immediate and sustained visual improvements achieved with PCL [30, 31]. This material's ability to maintain dermal volume and elasticity effectively over a longer duration might contribute to higher perceived outcomes among patients [32]. The slow degradation rate of PCL also results in fewer intervention cycles, reducing the frequency of follow‐up treatments, which may contribute further to patient contentment.

In this context, safety remains a critical consideration in the comparative evaluation of PCL and PLLA injections. The safety profile of both materials was favorable, with no statistically significant differences in terms of injection site reactions, nodule formation, discoloration, infection, or adverse events like granulomas, vascular occlusion, hypersensitivity reactions, and bruising. Both materials, therefore, are considered safe for use in NLF correction when administered by trained professionals following stringent protocols. However, the slight numerical differences observed in the occurrence of some adverse events warrant further investigation, particularly in larger and more diverse cohorts.

The observed efficacy and safety of PCL and PLLA can also be contextualized within the broader scope of their material properties. The viscoelasticity of PCL provides a unique advantage in maintaining tissue support and integrity over extended periods [33]. Its mechanical strength helps withstand dynamic facial movements; thereby preserving the aesthetic correction longer [34, 35]. On the other hand, PLLA's primary utility lies in its capacity to induce neocollagenesis, which, although effective, manifests more gradually and diminishes faster than PCL due to its quicker resorption [36]. Furthermore, PCL has higher viscoelasticity, requiring a 25G needle to ensure smooth injection; PLLA has smaller particles, and a 27G needle reduces trauma and facilitates even distribution. Despite the different technical parameters, PCL shows significantly better long‐term outcomes than PLLA, which may be related to its degradation kinetics and collagen‐stimulating capacity.

These findings also underscore the importance of personalized treatment approaches in cosmetic dermatology. Selecting the appropriate material based on patient‐specific factors such as desired longevity of results, potential for adverse reactions, and individual skin characteristics can optimize clinical outcomes. For patients seeking long‐term correction with minimal interventions, PCL may be the more suitable choice. Conversely, for those who prioritize biostimulatory effects with a shorter temporal horizon, PLLA could be preferential.

Moreover, the differences in procedure duration and recovery time noted between the two groups were not statistically significant, suggesting that from an operational standpoint, both materials offer similar practical benefits in clinical settings. Although the slightly shorter return‐to‐work time in the PCL group was not significantly different, the trend might imply a marginal edge in terms of patient convenience and post‐procedural downtime, albeit not overwhelmingly conclusive.

The study is not without limitations. The retrospective design inherently carries potential biases, and the relatively small sample size may limit the generalizability of the results. Future research should aim at larger, multi‐center trials with longer follow‐up periods to validate these findings. Additionally, exploring the molecular and histopathological changes induced by PCL and PLLA will provide deeper insights into their mechanisms of action and long‐term effects on skin architecture and collagen dynamics.

## 5 Conclusion

In conclusion, the comparative analysis of PCL and PLLA injections for NLF correction reveals that while both materials are effective and safe, PCL demonstrates superior long‐term efficacy in reducing wrinkle severity and enhancing aesthetic appearance, possibly due to its prolonged biodegradation and sustained collagen‐stimulating effects. These findings emphasize the value of material selection tailored to patient‐specific needs and aesthetic goals in optimizing outcomes in cosmetic dermatology. Future studies focusing on mechanistic aspects and broader patient populations will further refine the clinical application of these injectable materials.

## Author Contributions

Shanqing Wang: study design, data analysis, drafting the manuscript and revision of the manuscript. Kaixuan Hu: data collection and analysis, drafting the manuscript, investigation. All authors read and approved the final version of the manuscript.

## Ethics Statement

This study was approved by the Ethics Committee of Quzhou Kecheng Dainier Plastic Surgery Outpatient in accordance with regulatory and ethical guidelines pertaining to retrospective research studies and conducted in accordance with the Declaration of Helsinki.

## Consent

Informed consent was waived for this retrospective study due to the exclusive use of de‐identified patient data, which posed no potential harm or impact on patient care.

## Conflicts of Interest

The authors declare no conflicts of interest.

## Data Availability

All data generated or analyzed in this study are included in the present manuscript.
